# Days at Home After Hip Fracture Among Older Adults With and Without Dementia

**DOI:** 10.1001/jamanetworkopen.2026.18658

**Published:** 2026-06-16

**Authors:** Rebecca Rodin, Alexander K. Smith, Siqi Gan, Edie Espejo, John Boscardin, Lauren J. Hunt, Carmen E. Quatman, R. Sean Morrison

**Affiliations:** 1 Brookdale Department of Geriatrics and Palliative Medicine, Icahn School of Medicine at Mount Sinai (ISMMS), New York, New York; 2 Division of Geriatrics, University of California, San Francisco; 3 Department of Epidemiology and Biostatics, University of California, San Francisco; 4 Northern California Institute for Research and Education, San Francisco; 5 Philip R. Lee Institute for Health Policy Studies, University of California, San Francisco; 6 Departments of Emergency Medicine and Orthopaedic Surgery, The Ohio State University Wexner Medical Center, Columbus; 7 J.J. Peters VA Medical Center, Bronx, New York

## Abstract

**Importance:**

Hip fracture is common among older adults and is associated with considerable morbidity and mortality; it is nearly twice as common in those with dementia, who may also experience worse postfracture outcomes. Time spent at home is an important quality-of-life indicator, but this outcome has not been previously examined following hip fracture in older adults with and without dementia.

**Objective:**

To compare days at home and survival for the year after hip fracture among older adults with and without dementia and to identify factors associated with fewer days at home among those with dementia.

**Design, Setting, and Participants:**

This longitudinal cohort study used national administrative data from 100% of Medicare beneficiaries from 2012 to 2021 to identify community-dwelling older adults (aged ≥65 years) with and without dementia hospitalized for a hip fracture. Data were analyzed from January 1, 2012, to December 31, 2021.

**Exposure:**

Hip fracture hospitalization.

**Main Outcomes and Measures:**

Days at home and survival at 30 days, 6 months, and 1 year after hip fracture.

**Results:**

Among 1 756 388 Medicare beneficiaries hospitalized for hip fracture, the mean [SD] age was 82.5 [8.1] years, 1 237 193 (70.4%) were female, 65 889 [3.8%] were Black or African American, 93 362 [5.3%] were Hispanic, 1 547 090 [88.1%] were non-Hispanic White, and 513 698 (29.2%) had dementia. In the year following hip fracture, older adults with dementia died 50.3 days earlier than those without dementia (adjusted mean [SD] days, 264.6 [143.2] vs 314.9 [107.3]). Those with dementia who survived 1 year after fracture had 53.9 fewer days at home compared with those without dementia (adjusted mean [SD] days, 263.8 [129.5] vs 317.7 [72.9]) due to more time in skilled nursing (adjusted mean [SD] days, 38.2 [55.8] vs 24.2 [37.4]) and long-term care facilities (adjusted mean [SD] days, 52.5 [111.5] vs 12.4 [53.7]). Findings were similar at 1 and 6 months. Among individuals with dementia, Medicaid eligibility, rural residence, geographic region, and being aged 85 years or older were associated with the fewest days at home at 1 year.

**Conclusions and Relevance:**

In this cohort study, older adults with dementia had shortened survival and more time in skilled nursing or long-term care facilities after hip fracture than those without dementia. Differences in days at home were associated with structural and socioeconomic factors among those with dementia, with implications for health system policy, prognostic counseling, and discussions about long-term care needs in this population.

## Key Points

**Question:**

Do older adults with dementia spend less time at home after hip fracture than those without dementia?

**Findings:**

In this cohort study of over 1 million community-dwelling older adults with hip fracture, those with dementia lived 50.3 fewer days and those with dementia who survived 1 year after hip fracture spent 53.9 fewer days at home compared with adults with no dementia.

**Meaning:**

These findings can inform prognostic counseling, care planning, and decisions about rehabilitation, care intensity, and allocation of supportive services.

## Teaser

This cohort study evaluates days at home and survival for the year after hip fracture among older adults with and without dementia.

## Introduction

Hip fracture is a common and life-altering event in older adults and nearly twice as frequent in those with dementia.^1^ It is associated with high mortality, functional decline, and roughly $10 billion in annual US health care expenditures.^2,3,4^ Yet, postfracture outcomes are heterogenous, ranging from return to home, prolonged institutionalization, or death.^5^ Individuals with dementia may be particularly vulnerable to these adverse postfracture outcomes,^2,6^ yet risk factors for these outcomes or measures to circumvent them have not been adequately identified.

Days at home is a validated patient-centered outcome that captures time spent outside institutional settings.^5,7,8^ Knowledge of the probability of returning home is critical for care-planning discussions, decisions about pursuing prolonged and potentially futile postfracture rehabilitation, and mobilization of resources to help patients remain at home or transition to long-term care (LTC). Few studies have examined days at home after hip fracture^9,10^ or factors associated with variation in this outcome. One US study found an association of depression with days at home after hip fracture, although this association was attenuated after accounting for chronic disease burden.^11^ Importantly, prior studies of days at home after hip fracture have largely excluded the 50% of Medicare beneficiaries enrolled in Medicare Advantage (MA)^5,11,12^ and have rarely focused on individuals with dementia.^5^

We used 100% Medicare claims for fee-for-service and MA beneficiaries to compare days at home and survival after hip fracture among older adults with and without dementia. We sought to identify factors associated with risk for spending fewer days at home for people with dementia to inform postfracture prognostic and care planning discussions and health system policies and quality measurement. The latter is particularly relevant to value-based payment models for hip fracture that increasingly incorporate postacute outcomes, such as rehospitalizations.

## Methods

### Study Design

In this longitudinal cohort study, we identified community-dwelling older adults (aged 65 years or older) with and without dementia hospitalized for hip fracture between January 1, 2013, and December 31, 2020, with a look-back period of 1 year and a follow-up period up to 1 year, or until death if occurring first, starting from the date of hospital admission. We used Medicare claims data (2012-2021) from the Centers for Medicare & Medicaid Services (CMS) for 100% of Medicare beneficiaries using the Master Beneficiary Summary File (MBSF), Medicare Provider and Analysis Review (MedPAR), Outpatient, Carrier, Skilled Nursing Facility (SNF), Minimum Data Set (MDS), Durable Medical Equipment, Home Health, and Hospice files.

This study was approved by the institutional review boards of The Icahn School of Medicine at Mount Sinai and University of California, San Francisco with informed consent exemptions as the research involved no more than minimal risk and could not be carried out without a waiver. The study adhered to the Strengthening the Reporting of Observational Studies in Epidemiology (STROBE) reporting guidelines for cohort studies.

### Sample

We used the MBSF and MedPAR files to identify older (aged ≥65 years) beneficiaries with fee-for-service Medicare and MA with and without dementia hospitalized for hip fracture. We excluded individuals not residing at home (ie, in a care facility) within 1 day of their fracture because we were interested in the outcomes of hip fracture on community-dwelling older adults. LTC residence was determined using a validated method that uses both Part A and MDS data.^13^ Dementia status was determined using a validated method (sensitivity, 79% and specificity, 88%) employing *International Classification of Diseases, Ninth Revision (ICD-9) *and *Tenth Revision (ICD-10)* diagnosis codes.^14,15^

Hip fractures were identified using the MedPAR file and an established list of *ICD-9* and *ICD-10* diagnosis codes.^16^ We excluded codes for pathologic fractures and those considered late effects from a prior fracture. All *ICD-9* codes were converted to *ICD-10* codes and reviewed by one of the authors (C.Q.), an orthopedic surgeon (see eAppendix 1 in Supplement 1). Hip fractures were further classified based on anatomic location and surgical procedure type, which were also reviewed by C.Q. (see eAppendix 2 in Supplement 1). For participants with multiple hip fractures, only the first occurring fracture was included as the index hospitalization.^5^

### Outcomes

We measured days at home—days spent alive at home rather than in a hospital, inpatient rehabilitation facility, psychiatric facility, SNF, or LTC (ie, nursing home) facility—at 30 days, 6 months (secondary outcomes), and 1 year (primary outcome) after hip fracture.^17,18^ The minimum clinically important differences (MCIDs) for days at home are 3 days in a 30-day period,^19^ 8 to 14 days in a 6-month period,^20^ and 18.6 days in a 1-year period.^8^

We measured days at home as a continuous variable, defined as total days alive in the follow-up period minus days spent in inpatient hospitals, rehabilitation facilities, psychiatric facilities, SNFs, or LTC facilities (hereafter called facilities). Emergency department (ED) visits and observation stays were not measured because our sample included MA beneficiaries, for whom these encounters rely on data that were not released by CMS before 2015 and remained incomplete until 2018.^21,22^

#### Secondary Outcomes

Survival and place of death were secondary outcomes. Overall survival within 1 year after hip fracture was first assessed for the entire cohort, adjusted for age and sex, and stratified by dementia status. We then calculated mean survival among participants who died within specific time intervals: 0 to 30 days, 31 days to 6 months, or more than 6 months up to 1 year after hip fracture. Participants who died in an earlier interval were not included in later intervals (eg, those who died within 30 days after hip fracture were excluded from the 31-day to 6-month and >6-month groups). We recorded place of death for decedents to better understand care settings, intensity of health care utilization, and treatment goals at end-of-life following hip fracture, such as whether participants enrolled in hospice (see eAppendix 3 in Supplement 1).

### Covariates

We used the Andersen behavioral model to identify covariates, which were organized into the 3 domains postulated to influence health services utilization^23,24^: predisposing factors (eg, social and demographic structures), enabling factors that assist people to use services (eg, availability of services), and need factors that motivate use (eg, chronic diseases). These variables are listed in Table 1. Race was assessed using the Research Triangle Institute variable in the MBSF file to describe the sample and because it reflects social and structural factors that influence access to care and postacute recovery trajectories.^25^ The race variable was created by taking the beneficiary race code that has historically been used by the Social Security Administration (and is in turn used in CMS’s enrollment data base) and applying an algorithm that identifies more beneficiaries as Hispanic or Asian. Hospital size (bed count) and regional SNF and LTC availability (number of SNF and LTC beds per hospital referral region) data were obtained from linked American Hospital Association files. Geographic region was based on the 9 US census divisions using the MBSF file. Rurality (urban vs rural) was determined using the 2013 National Center for Health Classification Scheme.^26^ An Elixhauser comorbidity index was calculated using a 1-year lookback period from MedPAR inpatient claims so as to include MA beneficiaries^27,28,29^; outpatient and carrier claims were additionally incorporated in sensitivity analyses restricted to fee-for-service beneficiaries to ensure comorbidities were not undercounted.^30^ Delirium was determined using a validated list of *ICD-9* and *ICD-10* diagnosis codes during the index hospitalization (see eAppendix 4 in Supplement 1).^31^

**Table 1.** Baseline Characteristics of Older Adults With and Without Dementia Hospitalized for Hip Fracture

Characteristic	Patients, No. (%)		
	Total (n = 1 756 388)	Dementia (n = 513 698)	No dementia (n = 1 242 690)
Age, y			
Mean (SD)	82.5 (8.1)	85.6 (7.0)	81.3 (8.2)
Median (IQR)	83 (76-89)	86 (81-91)	82 (75-88)
<75	338 910 (19.3)	38 533 (7.5)	300 377 (24.2)
75-84	640 401 (36.5)	168 462 (32.8)	471 939 (38.0)
≥85	777 077 (44.2)	306 703 (59.7)	470 374 (37.9)
Sex			
Female	1 237 193 (70.4)	370 287 (72.1)	866 906 (69.8)
Male	519 146 (29.6)	143 403 (27.9)	375 743 (30.2)
Race and ethnicity			
American Indian or Alaska Native	7438 (0.4)	1966 (0.4)	5472 (0.4)
Asian or Pacific Islander	28 684 (1.6)	8773 (1.7)	19 911 (1.6)
Black or African American	65 889 (3.8)	24 460 (4.8)	41 429 (3.3)
Hispanic	93 362 (5.3)	29 951 (5.8)	63 411 (5.1)
Non-Hispanic White	1 547 090 (88.1)	445 504 (86.7)	1 101 586 (88.7)
Unknown	6030 (0.3)	837 (0.2)	5193 (0.4)
Other^a^	7846 (0.5)	2199 (0.4)	5647 (0.5)
Geographic region			
East North Central	260 101 (14.8)	74 612 (14.5)	185 489 (14.9)
East South Central	135 230 (7.7)	39 190 (7.6)	96 040 (7.7)
Mid-Atlantic	228 582 (13.0)	65 517 (12.8)	163 065 (13.1)
Mountain	121 649 (6.9)	33 099 (6.4)	88 550 (7.1)
New England	84 209 (4.8)	24 040 (4.7)	60 169 (4.8)
Pacific	230 227 (13.1)	69 834 (13.6)	160 393 (12.9)
South Atlantic	372 105 (21.2)	116 277 (22.6)	255 828 (20.6)
West North Central	125 690 (7.2)	33 012 (6.4)	92 678 (7.5)
West South Central	198 066 (11.3)	58 010 (11.3)	140 056 (11.3)
Rurality			
Rural	326 186 (18.6)	87 122 (17.0)	239 064 (19.2)
Urban or suburban	1 417 169 (80.7)	423 267 (82.4)	993 902 (80.0)
Medicare Advantage enrollment	595 146 (33.9)	161 758 (31.5)	433 388 (34.9)
Frailty, mean (SD)			
Q1 (0.03-0.18)	439 113 (25.0)	11 989 (2.3)	427 124 (34.4)
Q2 (0.18-0.22)	439 133 (25.0)	73 866 (14.4)	365 267 (29.4)
Q3 (0.22-0.28)	439 050 (25.0)	163 527 (31.8)	275 523 (22.2)
Q4 (0.28-0.74)	439 092 (25.0)	264 316 (51.5)	174 776 (14.1)
Delirium during admission	159 527 (9.1)	76 459 (14.9)	83 068 (6.7)
Fracture location			
Femoral neck	703 370 (40.1)	206 411 (40.2)	496 959 (40.0)
Intertrochanteric	832 498 (47.4)	246 276 (47.9)	586 222 (47.2)
Subtrochanteric	73 033 (4.2)	16 882 (3.3)	56 151 (4.5)
Unspecified	147 487 (8.4)	44 129 (8.6)	103 358 (8.3)
Surgical procedure			
Total arthroplasty	93 321 (5.3)	13 394 (2.6)	79 927 (6.4)
Hemiarthroplasty	526 006 (30.0)	167 158 (32.5)	358 848 (28.9)
Internal fixation or open reduction	870 445 (49.6)	250 556 (48.8)	619 889 (49.9)
Nonoperative	266 616 (15.2)	82 590 (16.1)	184 026 (14.8)
Decedents^b^	454 112 (25.9)	214 699 (41.8)	239 413 (19.3)

Abbreviation: Q, quartile.

^a^ Other is an official category contained within the Master Beneficiary Summary File; it is not known what groups are included in other.

^b^ Includes all participants who died within 1 year after hip fracture.

### Statistical Analysis

For descriptive data, we calculated frequencies with percentages for categorical variables and means with SDs or medians with IQRs for continuous variables. Overall survival up to 1 year was estimated using regression-based standardization with adjustment for age and sex. Unadjusted survival was visualized using Kaplan-Meier survival curves.

We compared the primary outcome, days at home, between those with and without dementia using unadjusted and adjusted analyses. Adjusted models used regression-based marginal standardization^32^ and included age, sex, comorbidities, hospital size, and regional SNF/LTC availability, consistent with prior studies.^11,33^ The primary days at home analyses were restricted to participants who survived the full interval of interest (30 days, 6 months, or 1 year). Participants who subsequently died were included in the analyses at earlier time points if they remained alive throughout the interval of interest (eg, included in the 30-day analysis if alive at 30 days).

We conducted secondary sensitivity analyses among participants who died during follow-up to describe patterns of time spent at home before death. To account for variation in days alive among decedents, days at home was expressed as a proportion: (total days alive minus days spent in facilities) divided by total days alive. Results among decedents were interpreted cautiously because no validated MCIDs exist for the proportion of days alive spent at home.

We identified factors associated with risk for having fewer days at home using multivariable linear regression models for those with dementia who survived 1-year after hip fracture. Models were built to identify the primary factors that may explain the association between dementia and days at home, with covariates clustered into the domains of the Andersen model.

We conducted additional sensitivity analyses for the primary days at home outcome and the multivariable regression model by excluding MA beneficiaries because certain measures relying on the Outpatient and Carrier files, such as procedure type, may be underdetected in these populations. We do not report *P* values or confidence intervals due to their limited utility in large samples (see eAppendix 5 in Supplement 1).^34^ Data were analyzed from January 1, 2012, to December 31, 2021 using SAS, version 9.4 (SAS Institute), Stata version 19 (StataCorp), and R version 4.4.1 (R Project for Statistical Computing.

## Results

We identified 1 756 388 community-dwelling older adults who were hospitalized for hip fracture (mean [SD] age, 82.5 [8.1] years; 1 237 193 [70.4%] female; 65 889 [3.8%] Black or African American, 93 362 [5.3%] Hispanic, and 1 547 090 [88.1%] non-Hispanic White), of whom 513 698 (29.2%) had dementia. Among the 454 112 participants who died within 1 year after fracture, 212 699 (47.3%) had dementia, representing 41.4% and 19.4% of all participants with and without dementia, respectively. Participant characteristics stratified by dementia status are in Table 1.

### Survival and Place of Death

**Figure 1.**
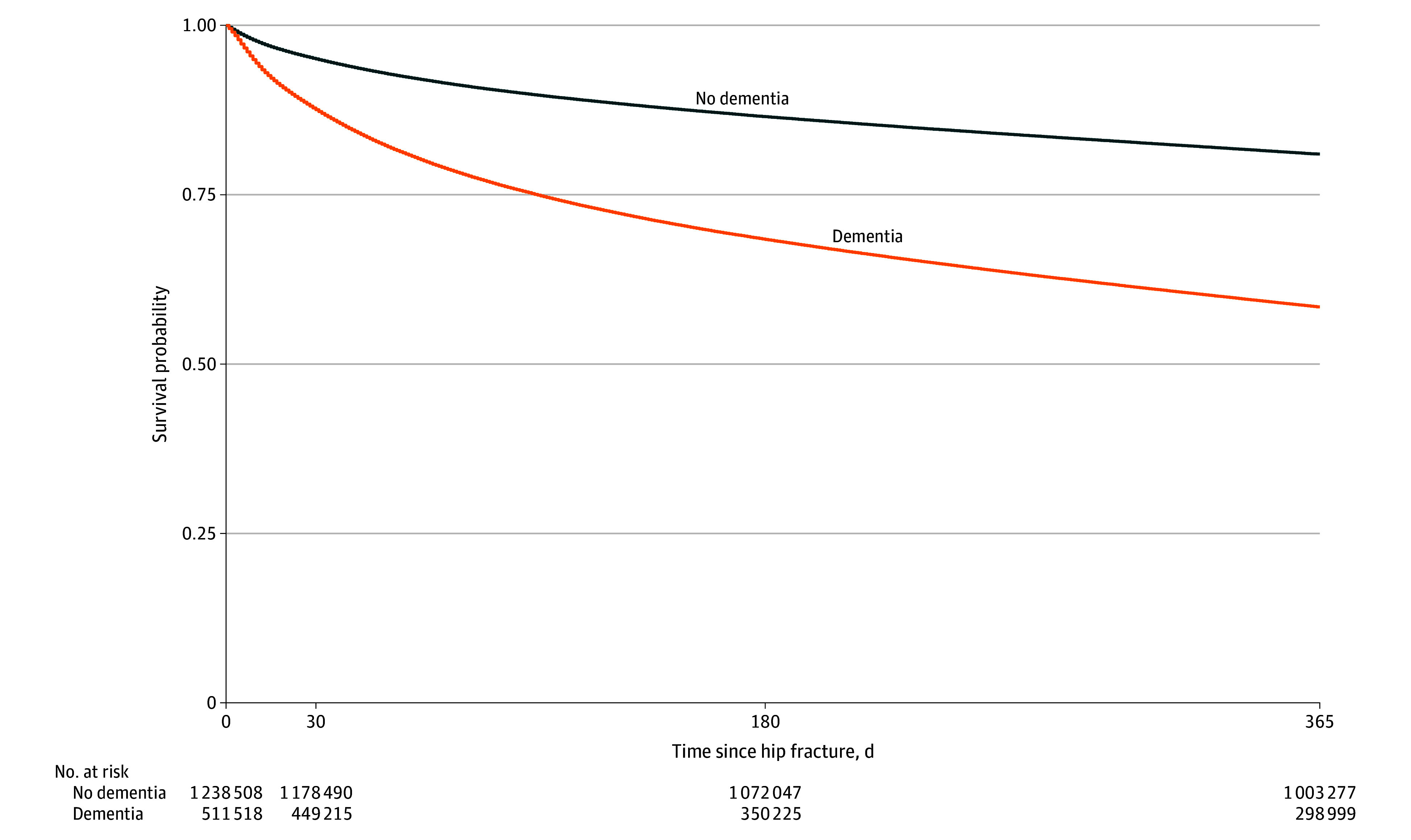
Kaplan-Meier Survival Curves for 1-Year Survival After Hip Fracture Hospitalization Among Older Adults With and Without Dementia

In adjusted analyses at 1 year, older adults with dementia survived 264.6 days, and those without dementia survived 314.9 days (adjusted mean difference, 50.3 days). Comparable survival differences were also observed for those who died within 30 days (adjusted mean, 28.0 [6.5] days vs 29.0 [4.5] days; difference, 1.0 day), 1 to 6 months (adjusted mean, 146.0 [64.7] days vs 165.0 [46.2] days; difference, 19.0 days), and 6 months to 1 year (adjusted mean: 264.6 [143.2] days vs 314.9 [107.3] days; difference, 50.3 days). Unadjusted survival is shown in Figure 1.

**Figure 2.**
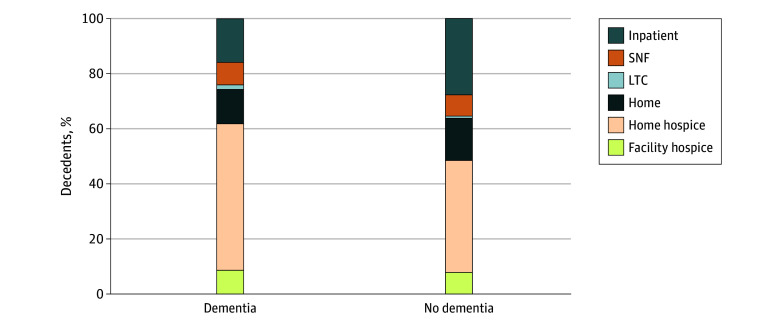
Bar Chart Showing Place of Death Among Hip Fracture Decedents, Stratified by Dementia Status Inpatient includes inpatient hospital, inpatient rehabilitation facility, and inpatient psychiatric facility. LTC indicates long-term care (ie, nursing home); SNF indicates skilled nursing facility.

The most common place of death was home hospice for those with and without dementia (239 413 [53.2%] and 214 699 participants [40.7%], respectively) (Figure 2). A greater proportion of decedents with dementia compared with those without dementia died in an inpatient hospice facility (18 446 [8.6%] vs 18 737 participants [7.8%]), SNF (17 584 [8.2%] vs 18 372 participants [7.7%]), or LTC facility (3402 [1.6%] vs 1948 [0.8%]). Inpatient hospital deaths were more common among those without dementia (660 468 [27.6%] vs 34 008 participants [14.0%]) (Figure 2).

### Days at Home

In the year following fracture, surviving older adults with dementia had 53.9 fewer days at home than those without dementia (adjusted mean [SD]: 263.8 [129.5] vs 317.7 [72.9] days), with differences associated with greater time in SNF (adjusted mean: 38.2 [55.8] days vs 24.2 [37.4] days; difference, 14.0 days) and LTC facilities (adjusted mean: 52.5 [111.5] days vs 12.4 [53.7] days; difference, 40.1 days) (Table 2; eFigure in Supplement 1). Days spent in other facilities that year were equivalent between groups (Table 2; eFigure in Supplement 1). Similar trends were observed at 30 days and 6 months following fracture (Table 2; eFigure in Supplement1) and in sensitivity analyses restricted to fee-for-service beneficiaries (eTable 1 in Supplement 1). In sensitivity analyses among decedents, those with dementia compared with those without dementia spent proportionally more days at home at 30 days (adjusted mean proportion, 0.25 [0.30] vs 0.15 [0.25]; difference, 0.1), an equivalent proportion of days at home at 6 months (adjusted mean proportion, 0.47 [0.34] vs 0.48 [0.33]; difference, 0.01), and proportionally fewer days at home at 1 year (adjusted mean proportion: 0.62 [0.35] vs 0.70 [0.28]; difference, 0.08) (eTable 2 in Supplement 1).

**Table 2.** Days at Home at 30 Days, 6 Months, and 1 Year After Hip Fracture in Surviving Older Adults With and Without Dementia

Time since fracture	Inpatient days, mean (SD)	Inpatient rehab facility days, mean (SD)	Inpatient psychiatry days, mean (SD)	SNF days, mean (SD)	LTC facility days, mean (SD)	Days at home, mean (SD)
Unadjusted						
30 d						
No dementia (n = 1 178 490)	7.5 (6.2)	1.1 (4.0)	0.0	9.8 (10.9)	0.2 (2.0)	12.4 (11.1)
Dementia (n = 449 215)	7.2 (5.5)	0.6 (3.2)	0.0 (0.3)	13.4 (11.2)	0.8 (4.1)	8.6 (10.7)
6 mo						
No dementia (n = 1 070 760)	9.4 (9.4)	1.3 (4.6)	0.0 (0.6)	21.1 (30)	4.5 (23.1)	148.1 (41.4)
Dementia (n = 349 186)	9.3 (9.2)	0.8 (3.9)	0.1 (1.4)	34.7 (39.3)	21.2 (48.4)	117.7 (62.8)
1 y						
No dementia (n = 1 003 277)	10.6 (11.1)	1.4 (5.0)	0.0 (1.0)	23.2 (37.4)	11.6 (53.7)	319.5 (72.9)
Dementia (n = 298 999)	10.7 (11.1)	0.9 (4.3)	0.1 (1.8)	40.8 (55.8)	54.8 (111.5)	258.7 (129.5)
Adjusted^a^						
30 d						
No dementia (n = 1 178 490)	7.6	1.1	0.0	10.2	0.2	12.0
Dementia (n = 449 215)	7.1	0.6	0.0	12.7	0.7	9.4
6 mo						
No dementia (n = 1 070 760)	9.4	1.3	0.0	22.0	4.8	146.8
Dementia (n = 349 186)	9.2	0.8	0.1	32.5	20.3	121.0
1 y						
No dementia (n = 1 003 277)	10.7	1.4	0.0	24.2	12.4	317.7
Dementia (n = 298 999)	10.6	0.9	0.1	38.2	52.5	263.8

Abbreviations: LTC, long-term care; SNF, skilled nursing facility.

^a^ Adjusted for age, sex, hospital size, regional SNF and/or LTC bed availability, and comorbidities. Adjustment was performed using regression-based marginal standardization.

### Factors Associated With Days at Home for Older Adults With Dementia

Among older adults with dementia who survived 1 year after hip fracture, several factors were associated with clinically significant fewer days at home (ie, ≥18.6 days MCID threshold) in the multivariable regression models (Table 3). These included being aged 85 years or older compared with being younger than 75 years (fully adjusted mean change in days at home, −22.6 days), Medicaid eligibility (−74.6 days), and fee-for-service Medicare compared with MA (−25.4 days) (Table 3). Differences by race were observed, with more days at home among Hispanic (38.8 days) and Asian or Pacific Islander (24.0 days) individuals compared with non-Hispanic White individuals (Table 3).

**Table 3.** Multivariable Regression Model of Days at Home at 1-Year After Hip Fracture Among Surviving Older Adults With Dementia

Variable	DAH, unadjusted mean (SD)	Model 1^a^		Model 2^b^		Model 3^c^	
		Change in DAH, adjusted mean	DAH, adjusted mean	Change in DAH, adjusted mean	DAH, adjusted mean	Change in DAH, adjusted mean	DAH, adjusted mean
Age							
<75	267.8 (123.1)	[Reference]	267.2	[Reference]	274.8	[Reference]	274.1
75-84	264.9 (125.5)	−2.5	264.7	−10.3	264.5	−10.0	264.1
≥85	253.1 (132.8)	−13.8	253.3	−23.6	251.2	−22.6	251.5
Sex							
Male	263.4 (123.9)	[Reference]	262.2	[Reference]	258.7	[Reference]	259.0
Female	257.3 (131.1)	−4.5	257.7	−0.7	258.0	−1.2	257.8
Race							
American Indian or Alaska Native	258.2 (129.1)	0.8	256.9	17.9	272.3	18.2	272.6
Asian or Pacific Islander	274.2 (123.8)	19.0	275.2	24.2	278.6	24.0	278.4
Black or African American	257.4 (128.2)	0.3	256.5	15.0	269.4	14.8	269.3
Hispanic	287.5 (114.9)	31.0	287.2	38.8	293.2	38.8	293.2
Non-Hispanic White	256.1 (130.5)	[Reference]	256.2	[Reference]	254.4	[Reference]	254.4
Other	276.7 (118.7)	19.2	275.3	17.9	272.3	17.6	272.0
Unknown	268.1 (124.7)	8.1	264.3	22.5	276.9	22.1	276.5
Region							
East North Central	247.0 (133.5)	NA	NA	[Reference]	246.5	[Reference]	246.8
East South Central	261.4 (128.5)	NA	NA	13.3	259.8	13.0	259.7
Mid-Atlantic	241.7 (137.4)	NA	NA	−4.5	242.0	−4.9	241.8
Mountain	286.9 (112.4)	NA	NA	37.0	283.4	36.5	283.3
New England	232.6 (142.6)	NA	NA	−6.8	239.7	−7.1	239.7
Pacific	291.5 (108.1)	NA	NA	40.9	287.4	40.6	287.4
South Atlantic	263.2 (123.9)	NA	NA	17.0	263.5	16.7	263.4
West North Central	221.2 (147.1)	NA	NA	−22.4	224.1	−22.6	224.1
West South Central	259.4 (129.6)	NA	NA	11.1	257.6	10.8	257.6
Urban or rural							
Urban	263.6 (126.4)	NA	NA	[Reference]	261.6	[Reference]	261.7
Rural	231.6 (141.5)	NA	NA	−20.3	241.3	−20.6	241.0
Medicare Advantage enrollment							
No	249.4 (128.3)	NA	NA	[Reference]	250.1	[Reference]	250.5
Yes	280.4 (129.7)	NA	NA	26.7	276.8	25.4	275.9
Medicaid eligibility							
No	271.7 (119.8)	NA	NA	[Reference]	273.3	[Reference]	273.2
Yes	207.1 (151.7)	NA	NA	−75.2	198.1	−74.6	198.6
Elixhauser Comorbidity Score							
Q1 (−26 to 0)	264.1 (128.0)	NA	NA	NA	NA	[Reference]	263.2
Q2 (1 to 8)	260.9 (129.7)	NA	NA	NA	NA	−4.4	258.8
Q3 (9 to 15)	257.1 (130.3)	NA	NA	NA	NA	−6.6	256.6
Q4 (16 to 81)	252.2 (129.8)	NA	NA	NA	NA	−9.7	253.5
Delirium during admission							
No	259.2 (129.6)	NA	NA	NA	NA	[Reference]	258.8
Yes	255.2 (128.8)	NA	NA	NA	NA	−5.4	253.5
Fracture location							
Femoral neck	266.6 (126.5)	NA	NA	NA	NA	[Reference]	265.1
Intertrochanteric	253.9 (131.2)	NA	NA	NA	NA	−11.2	253.9
Subtrochanteric	248.6 (131.4)	NA	NA	NA	NA	−16.4	248.6
Unspecified	252.7 (131.2)	NA	NA	NA	NA	−12.0	253.1
Surgical procedure							
Total arthroplasty	278.9 (116.8)	NA	NA	NA	NA	[Reference]	269.4
Hemiarthroplasty	262.1 (128.6)	NA	NA	NA	NA	−13.4	256.0
Internal fixation or open reduction	252.5 (130.1)	NA	NA	NA	NA	−11.9	257.5
Nonoperative	269.8 (130.6)	NA	NA	NA	NA	−5.9	263.5

Abbreviations: DAH, days at home; NA, not applicable; Q, quartile.

^a^ Model 1 means are adjusted for age, sex, and race.

^b^ Model 2 means are adjusted for all covariates in model 1 and insurance status (Medicare Advantage enrollment and Medicaid eligibility), geographic region, and rurality.

^c^ Model 3 means are adjusted for all covariates in model 2 and comorbidities, delirium during index admission, fracture type, and procedure type.

Geographic variation was also observed, including fewer days at home for rural compared with urban residence (−20.6 days). Those living in the West North Central region had fewer days at home (fully adjusted mean change in days at home: −22.6 days), whereas those living in the Pacific (40.6 days) and Mountain (36.5 days) regions had more days at home compared with the East North Central region (Table 3). Differences associated with comorbidity burden, delirium during index hospitalization, fracture type, and surgical procedure were smaller and did not exceed the MCID (Table 3).

In sensitivity analyses, similar trends were observed among decedents with dementia at 1 year, although no established MCIDs exist to determine their clinical significance (eTable 3 in Supplement 1). Findings were also similar in analyses restricted to fee-for-service beneficiaries, except that days at home was also associated with American Indian or Alaska Native race (fully adjusted mean change: 22.4 days) and residence in the South Atlantic region (18.6 days) (see eTable 4 in Supplement 1).

## Discussion

In this national cohort study of 1 756 388 community-dwelling older adults hospitalized with hip fracture, individuals with dementia compared with those without dementia had shortened survival by nearly 2 months, and those who survived spent approximately 2 fewer months at home in the year following injury, including 2 weeks more in SNFs and over 1 month more in LTC facilities. These findings highlight the increased risk of death and substantial burden of institutional care following hip fracture among older adults with dementia.

Among individuals with dementia, several factors were associated with clinically meaningful differences in days at home in multivariable models. Structural and socioeconomic factors, including insurance status, rural residence, and geographic region were associated with the largest differences in days at home and are potential targets for health system-level interventions. By contrast, differences associated with clinical factors, such as comorbidity burden, delirium, fracture type, and surgical procedure, were smaller and did not exceed established MCID thresholds.

The large differences associated with Medicaid eligibility and geographic context highlight the potential role of socioeconomic vulnerability and variation in access to community-based postacute care services (eg, inpatient rehabilitation, home health care, and other community-based supports) that affect return to and maintenance of community living.^35,36,37^ For example, Medicaid is the primary payer for long-term nursing home care, whereas Medicare generally does not cover long-term custodial care. As a result, individuals with Medicaid may be more likely to transfer from subacute to long-term care within the same facility. Differences by race may reflect variation in cultural values, caregiving structures, or social supports, although these factors could not be directly assessed. Future research should investigate the mechanisms underlying these differences and evaluate whether expanded home- and community-based services can improve time spent at home.

These findings add to the growing body of literature demonstrating that persons with dementia are at significantly higher risk for adverse outcomes than persons with intact cognition.^2,38^ By focusing on a clinically meaningful, patient-centered outcome, days at home, this study extends prior work by characterizing postfracture care patterns and identifying factors associated with variation in outcomes. These findings may help inform prognostic counseling and care planning discussions about expected postfracture outcomes, goals of care, anticipated rehabilitation, and the need for supportive services to remain at home or transition to LTC.

These findings also have implications for health system policy and value-based payment models, which increasingly incorporate postacute outcomes, such as readmission rates, to evaluate health system performance. Limited adjustment for dementia and structural factors may disadvantage institutions caring for more complex populations.^39^ By characterizing days at home in older adults with and without dementia, our study provides data to inform more equitable risk adjustment and benchmarking in value-based payment models.

This is the first study to describe place of death among older adults after hip fracture. Individuals with dementia were more likely to die in nursing (SNF and LTC) facilities or in hospice, consistent with greater time spent in these nursing settings and use of comfort-focused care. Conversely, individuals without dementia may be more likely to seek hospitalization for postfracture complications, which may contribute to their higher rates of in-hospital deaths. Nevertheless, the majority of older adults in both groups died at home. These trends resemble those reported for other serious or high-mortality conditions, such as cancer, for which nearly 40% of patients reportedly die at home.^40^ These findings underscore the importance of supporting home-based and hospice services for older adults with dementia following hip fracture to align care and patient preferences at the end of life.

In sensitivity analyses among decedents, patterns of time spent at home differed by survival duration, suggesting that days at home may vary over the course of illness. However, these findings should be interpreted cautiously, as no established MCID exists for these proportional measures, and they highlight the challenges of comparing outcomes between survivors and decedents.

### Limitations

Strengths of this study are the inclusion of the entire population of Medicare beneficiaries, including those enrolled in MA, who have historically been excluded from most studies.^5,41^ MA currently represents over half of all Medicare beneficiaries and is expected to continue to grow, although few studies to date have accounted for this expanding population.

Limitations include use of administrative data, which precluded assessment of functional status, symptom burden, dementia severity, and social supports, which may affect postfracture outcomes and quality of time at home. To be sure, not every day at home is a good day, and there may be tradeoffs between the benefits of remaining at home and increased caregiver burden, particularly for those with dementia.^42^ It is also possible that individuals may have remained at home due to financial constraints or limited engagement with the health system rather than true functional ability, which may affect the interpretation of our findings. Additionally, misclassification of dementia and exclusion of ED and observational stays may underestimate our findings and bias results toward the null.

## Conclusions

In this national cohort study of community-dwelling Medicare beneficiaries with hip fracture, dementia was associated with shorter survival, fewer days at home, and more time spent in SNF and LTC facilities. For those with dementia, Medicaid enrollment and residence in rural or West North Central regions were associated with the fewest number of days at home. These findings may help to inform health system and policy improvements, as well as discussions with patients and families about their goals, values, treatment preferences, and future care planning in this population.

## Notes

Supplement 1. eAppendix 1. Identification Hip Fracture Hospitalization Based on International Classification of Disease (ICD) Codes eAppendix 2. Classification of Hip Fractures Using ICD and Current Procedural Terminology (CPT) Codes eAppendix 3. Determination of Place of Death eAppendix 4. Identification of Delirium Using ICD Codes eAppendix 5. Additional Detail on Reporting Measures of Uncertainty eFigure. Heat Maps of Days at Home in the Year After Hip Fracture in Random 10 000 Person Samples of Older Adults With and Without Dementia eTable 1. Days at Home at 30 Days, 6 Months, and 1 Year After Hip Fracture in Older Adults With and Without Dementia, Restricted to Fee-For-Service Beneficiaries eTable 2. Proportion of Days Alive at Home at 30 Days, 6 Months, and 1 Year After Hip Fracture Among Decedents With and Without Dementia eTable 3. Multivariable Regression Model of Proportion of Days Alive at Home at 1-Year After Hip Fracture Among Decedents With Dementia eTable 4. Sensitivity Analysis of Multivariable Regression Model of Days Alive at Home at 1-Year After Hip Fracture Among Older Adults With Dementia, Restricted to Fee-For-Service Beneficiaries eReferences.

Supplement 2. Data Sharing Statement
