# Supplementary material for: Days at Home After Hip Fracture Among Older Adults With and Without Dementia
Source: JAMA Netw Open. 2026 Jun 16;9(6):e2618658. doi: 10.1001/jamanetworkopen.2026.18658 (PMC13273490; doi:10.1001/jamanetworkopen.2026.18658)
Supplement: Supplement 1. — eAppendix 1. Identification Hip Fracture Hospitalization Based on International Classification of Disease (ICD) Codes eAppendix 2. Classification of Hip Fractures Using ICD and Current Procedural Terminology (CPT) Codes eAppendix 3. Determination of Place of Death eAppendix 4. Identification of Delirium Using ICD Codes eAppendix 5. Additional Detail on Reporting Measures of Uncertainty eFigure. Heat Maps of Days at Home in the Year After Hip Fracture in Random 10 000 Person Samples of Older Adults With and Without Dementia eTable 1. Days at Home at 30 Days, 6 Months, and 1 Year After Hip Fracture in Older Adults With and Without Dementia, Restricted to Fee-For-Service Beneficiaries eTable 2. Proportion of Days Alive at Home at 30 Days, 6 Months, and 1 Year After Hip Fracture Among Decedents With and Without Dementia eTable 3. Multivariable Regression Model of Proportion of Days Alive at Home at 1-Year After Hip Fracture Among Decedents With Dementia eTable 4. Sensitivity Analysis of Multivariable Regression Model of Days Alive at Home at 1-Year After Hip Fracture Among Older Adults With Dementia, Restricted to Fee-For-Service Beneficiaries eReferences. [file jamanetwopen-e2618658-s001.pdf]

Supplemental Online Content

Rodin R, Smith AK, Gan S, et al. Days at home after hip fracture among older adults with and without dementia. *JAMA Netw Open*. 2026;9(6):e2618658.
doi:10.1001/jamanetworkopen.2026.18658

eAppendix 1. Identification Hip Fracture Hospitalization Based on International Classification of Disease (ICD) Codes
eAppendix 2. Classification of Hip Fractures Using ICD and Current Procedural Terminology (CPT) Codes
eAppendix 3. Determination of Place of Death
eAppendix 4. Identification of Delirium Using ICD Codes
eAppendix 5. Additional Detail on Reporting Measures of Uncertainty
eFigure. Heat Maps of Days at Home in the Year After Hip Fracture in Random 10 000 Person Samples of Older Adults With and Without Dementia
eTable 1. Days at Home at 30 Days, 6 Months, and 1 Year After Hip Fracture in Older Adults With and Without Dementia, Restricted to Fee-For-Service Beneficiaries
eTable 2. Proportion of Days Alive at Home at 30 Days, 6 Months, and 1 Year After Hip Fracture Among Decedents With and Without Dementia
eTable 3. Multivariable Regression Model of Proportion of Days Alive at Home at 1-Year After Hip Fracture Among Decedents With Dementia
eTable 4. Sensitivity Analysis of Multivariable Regression Model of Days Alive at Home at 1-Year After Hip Fracture Among Older Adults With Dementia, Restricted to Fee-For-Service Beneficiaries
eReferences.

This supplemental material has been provided by the authors to give readers additional information about their work.

© 2026 Rodin R et al. *JAMA Network Open*.

eAppendix 1. Identification Hip Fracture Hospitalization Based on International Classification of Disease (ICD) Codes

Identification of Hip Fracture
ICD-9: Included hospital admissions with code 820.XX and excluded admissions that were considered late effects from a prior hip fracture (733.81, 733.82, 905.3, V540-V549). Diagnostic codes could be in any position in the claim.

ICD-10: Included hospital admissions with codes S72.0XX, S72.1XX, S72.2XX with letters A, B, or C to indicate initial encounter. Diagnostic codes were required to be in the first or first secondary position.

© 2026 Rodin R et al. *JAMA Network Open*.

eAppendix 2. Classification of Hip Fractures Using ICD and Current Procedural Terminology (CPT) Codes

Fracture Location

Fracture Location	ICD-9	ICD-10
Femoral Neck	820.00, 820.01, 820.02, 820.03, 820.09, 820.10, 820.11, 820.12, 820.13, 820.19	S72.0XX (with letters A, B or C)
Intertrochanteric	820.20, 820.21, 820.30, 820.31	S721.XX, S721.4X (with letters A, B, or C)
Subtrochanteric	820.22, 820.32	S72.2.2XX (with letters A, B or C)

* ICD-9 codes could be any position; ICD-10 codes were checked in the first or second positions.

Surgical Procedure Type

- Surgery was defined as the presence of ICD-9, ICD-10, or CPT procedural codes specified in the table below[1]
- Non-operative management was defined as a diagnostic code for hip fracture without any of the surgical ICD or CPT codes listed below

Surgical Procedure	CPT	ICD-9	ICD-10
Internal Fixation/Open Reduction	27235, 27236, 27244, 27245, 27248, 27269	78.55, 79.25, 79.35, 79.65	0QH604Z, 0QH604Z, 0QH644Z, 0QH704Z, 0QH734Z, 0QH744Z, 0QS604Z, 0QS634Z, 0QS644Z, 0QS704Z, 0QS734Z, 0QS744Z, 0SH934Z, 0SH944Z, 0SHB04Z, 0SHB34Z, 0SHB44Z, 0SS904Z, 0SS905Z, 0SS904Z, 0SSB04Z, 0SSB05Z, 0QH706Z, 0QH606Z, 0QH736Z, 0QH636Z
Hemiarthroplasty	27125	81.52	0SRC0JZ, 0SRD0JZ, 0SRR019, 0SRR01A, 0SRR01Z, 0SRR039, 0SRR03A, 0SRR03Z, 0SRR07Z, 0SRR0J9, 0SRR0JA, 0SRR0JZ, 0SRR0KZ, 0SRS019, 0SRS01A, 0SRS01Z, 0SRS039, 0SRS03A, 0SRS03Z, 0SRS07Z, 0SRS0J9, 0SRS0JA, 0SRS0JZ, 0SRS0KZ
Total Arthroplasty	27130	81.51	0SR90JZ, 0SRB0JZ, 0SRG0JZ, 0SR9019, 0SR901A, 0SR901Z, 0SR9029, 0SR902A, 0SR902Z, 0SR9039, 0SR903A, 0SR903Z, 0SR9049, 0SR904A, 0SR904Z, 0SR907Z, 0SR90J9, 0SR90JA, 0SR90JZ, 0SR90KZ, 0SRB019, 0SRB01A, 0SRB01Z, 0SRB029, 0SRB02A, 0SRB02Z, 0SRB039, 0SRB03A, 0SRB03Z, 0SRB049, 0SRB04A, 0SRB04Z, 0SRB07Z, 0SRB0J9, 0SRB0JA, 0SRB0JZ, 0SRB0KZ, 0SRA009, 0SRA00A, 0SRA00Z, 0SRA019, 0SRA01A, 0SRA01Z, 0SRA039, 0SRA03A, 0SRA03Z, 0SRA0J9, 0SRA0JA, 0SRA0JZ, 0SRE009, 0SRE00A, 0SRE00Z, 0SRE019, 0SRE01A, 0SRE01Z, 0SRE039, 0SRE03A, 0SRE03Z, 0SRE0J9, 0SRE0JA
Non-Operative	Absence of any of the above CPT or ICD codes		

* ICD-9 codes could be any position; ICD-10 codes were checked in the first or second positions.

© 2026 Rodin R et al. *JAMA Network Open*.

eAppendix 3. Determination of Place of Death

Death occurring in a facility setting was defined as having a claim showing admission to an inpatient hospital, inpatient rehabilitation, inpatient psychiatry, skilled nursing, or long-term care facility on the day before death. Death occurring at home was defined as the absence of any of the above claims on the day prior to death. Hospice enrollment was defined as the presence of a hospice claim on the date of death. These were further categorized as home or facility hospice, according to whether the participant's death occurred at home or in a facility setting, respectively, based on the approach described above.

© 2026 Rodin R et al. *JAMA Network Open*.

eAppendix 4. Identification of Delirium Using ICD Codes

Delirium during the index hip fracture hospitalization was determined using the following validated list of ICD-9 and ICD-10 codes:[2]

ICD-9: 293, 2931, 29281, 29011, 2903, 2904, 291, 2939, 78009, 29381, 29382, 29383, 29384, 29389, 293, 2931, 29281, 29011, 2903, 29041, 291, 2939, 78009, 29381, 29382, 29383, 29384, 29389, 29012, 29013, 29043, 29211, 29212, 2922, 78002, 2902, 29042, 2908, 2909, 292, 29282, 3483, 34831, 34839, 34982, 78097

ICD-10: F05, F10121, F10221, F10231, F10921, F11121, F11221, F11921, F12121, F12221, F12921, F13121, F13221, F13231, F13921, F13931, F14121, F14221, F14921, F15121, F15221, F15921, F16121, F16221, F16921, F18121, F18221, F18921, F19121, F19221, F19231, F19921, F19931, A812, E512, G0431, G0432, G0439, G92, G9340, G9341, G9349, I673, I674, I6783, J1081, J1181, P9160, P9161, P9162, P9163

© 2026 Rodin R et al. *JAMA Network Open*.

eAppendix 5. Additional Detail on Reporting Measures of Uncertainty

We do not report p values or confidence intervals. Although opinions vary about reporting measures of uncertainty with 100% population data,[3-5] we elected to omit them for readability and practicality. With the very large sample size in this study, nearly all *p* values were <0.001 and nearly all the endpoints of the confidence intervals did not differ from the estimates in the reported number of decimal places.

© 2026 Rodin R et al. *JAMA Network Open*.

eFigure. Heat Maps of Days at Home in the Year After Hip Fracture in Random 10 000 Person Samples of Older Adults With and Without Dementia

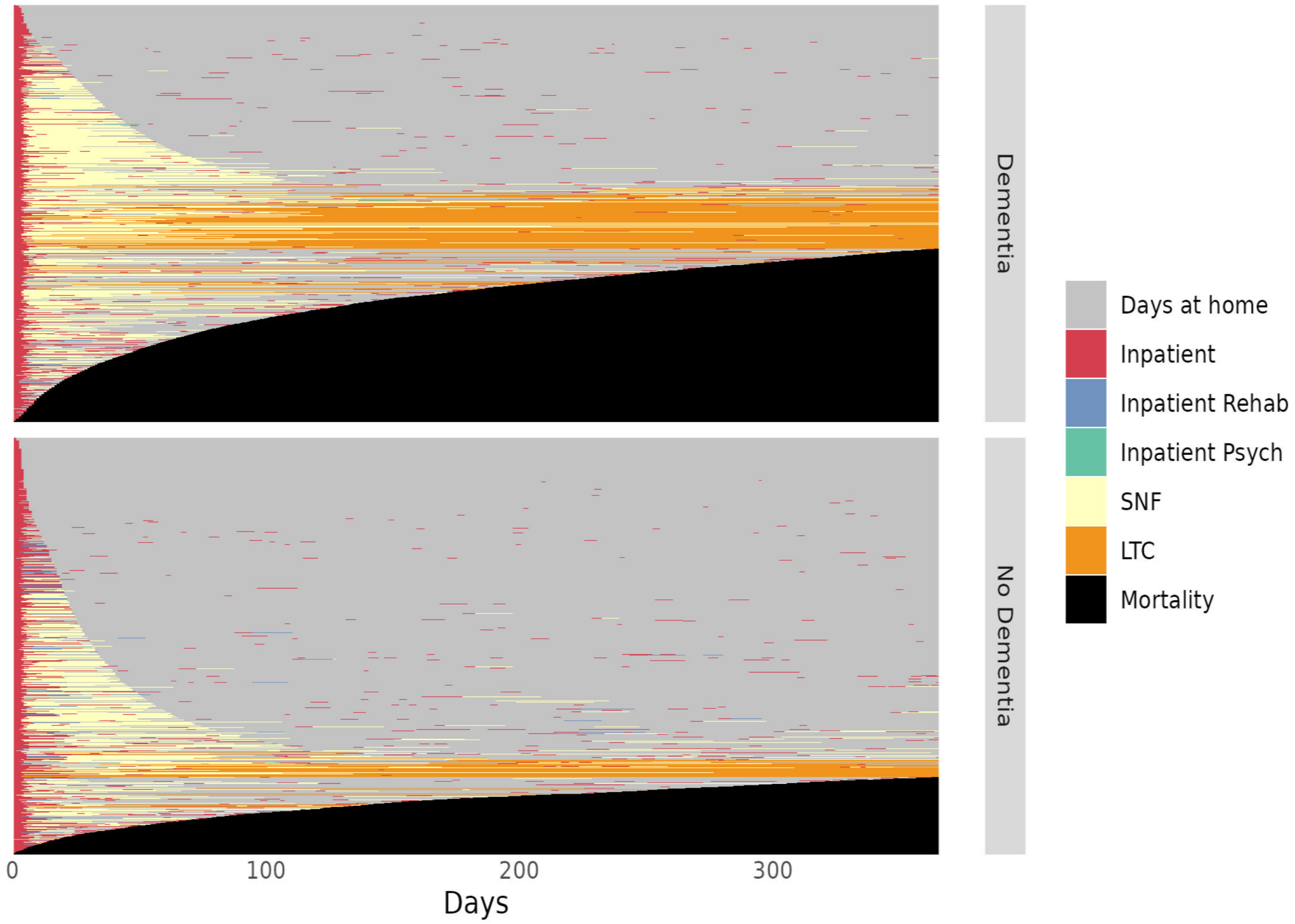

SNF = skilled nursing facility; Inpatient Psych = Inpatient Psychiatric Facility; Inpatient Rehab = Inpatient Rehabilitation Facility

© 2026 Rodin R et al. *JAMA Network Open*.

eTable 1. Days at Home at 30 Days, 6 Months, and 1 Year After Hip Fracture in Older Adults With and Without Dementia, Restricted to Fee-For-Service Beneficiaries

Time Since Fracture		Inpatient Days, mean (SD)	Inpatient Rehab Facility Days, mean (SD)	Inpatient Psychiatry Days, mean (SD)	SNF Days, mean (SD)	LTC Facility Days, mean (SD)	Days at Home, mean (SD)
**Unadjusted**							
**30 days**	No Dementia (N=749,278)	8.0 (6.6)	1.5 (4.6)	0.0 (0.1)	12.9 (10.9)	0.0 (0.8)	9.0 (10.2)
	Dementia (N=299,925)	7.4 (5.8)	0.8 (3.6)	0.0 (0.3)	17.2 (10.0)	0.2 (2.1)	5.2 (8.7)
**6 months**	No Dementia (N=680,213)	10.0 (9.9)	1.7 (5.3)	0.0 (0.7)	28.5 (32.7)	3.5 (19.0)	141.0 (42.2)
	Dementia (N=234,154)	9.7 (9.6)	1.1 (4.5)	0.1 (1.6)	45.3 (40.3)	17.9 (42.3)	110.1 (60.6)
**1 year**	No Dementia (N=636,715)	11.4 (11.7)	1.8 (5.7)	0.0 (1.1)	31.4 (41.5)	10.6 (49.9)	311.6 (74.3)
	Dementia (N=200,612)	11.1 (11.5)	1.2 (4.9)	0.1 (2.1)	53.1 (59.6)	51.0 (105.1)	249.7 (127.9)
**Adjusted for Age, Sex, Hospital Size, Regional SNF/LTC Bed Availability, and Comorbidities[a,b]**							
**30 days**	No Dementia (N=749,278)	8.1	1.4	0.0	13.3	0.0	8.6
	Dementia (N=299,925)	7.3	0.9	0.0	16.3	0.2	6.1
**6 months**	No Dementia (N=680,213)	10.1	1.7	0.0	29.5	3.8	139.6
	Dementia (N=234,154)	9.5	1.1	0.1	42.5	17.1	113.8
**1 year**	No Dementia (N=636,715)	11.4	1.8	0.0	32.5	11.4	309.7
	Dementia (N=200,612)	11.0	1.2	0.1	49.9	48.7	255.4

[a]Adjustment was performed using regression-based marginal standardization
[b]Elixhauser comorbidity index scores utilized MedPAR, Outpatient and Carrier files for this sensitivity analysis restricted to fee-for-service beneficiaries
SNF = skilled nursing facility; LTC = long-term care

© 2026 Rodin R et al. *JAMA Network Open*.

eTable 2. Proportion of Days Alive[a] at Home at 30 Days, 6 Months, and 1 Year[b] After Hip Fracture Among Decedents With and Without Dementia[c]

Time Since Fracture		Inpatient Days, mean (SD)	Inpatient Rehab Facility Days, mean (SD)	Inpatient Psychiatry Days, mean (SD)	SNF Days, mean (SD)	LTC Facility Days, mean (SD)	Days at Home, mean (SD)
**Unadjusted**							
**30 days**	No Dementia (N=64,200)	0.74 (0.30)	0.01 (0.08)	0.00 (0.00)	0.11 (0.22)	0.00 (0.00)	0.15 (0.25)
	Dementia (N=64,483)	0.62 (0.32)	0.00 (0.05)	0.00 (0.01)	0.12 (0.24)	0.00 (0.00)	0.25 (0.30)
**6 months**	No Dementia (N=107,730)	0.23 (0.20)	0.01 (0.06)	0.00 (0.00)	0.29 (0.30)	0.01 (0.09)	0.47 (0.33)
	Dementia (N=100,029)	0.17 (0.16)	0.01 (0.04)	0.00 (0.01)	0.32 (0.31)	0.02 (0.14)	0.48 (0.34)
**1 year**	No Dementia (N=67,483)	0.08 (0.07)	0.01 (0.02)	0.00 (0.00)	0.15 (0.18)	0.07 (0.20)	0.70 (0.28)
	Dementia (N=50,187)	0.06 (0.06)	0.00 (0.02)	0.00 (0.01)	0.17 (0.02)	0.16 (0.31)	0.62 (0.35)
**Adjusted for Age, Sex, Hospital Size, Regional SNF/LTC Bed Availability, and Comorbidities[d]**							
**30 days**	No Dementia (N=64,200)	0.73	0.01	0.00	0.11	0.00	0.15
	Dementia (N=64,483)	0.63	0.00	0.00	0.12	0.00	0.25
**6 months**	No Dementia (N=107,730)	0.22	0.01	0.00	0.30	0.01	0.47
	Dementia (N=100,029)	0.18	0.01	0.00	0.31	0.02	0.48
**1 year**	No Dementia (N=67,483)	0.08	0.01	0.00	0.15	0.07	0.70
	Dementia (N=50,187)	0.06	0.00	0.00	0.17	0.15	0.62

[a]To account for variation in survival duration among decedents, days at home was expressed as a proportion: (total days alive minus days spent in a facility) divided by total days alive
[b]Participants who died within 30 days of hip fracture were counted in the 30-day period, those who died between 31 days to 6 months were counted in the 6-month period; and those who died after 6 months were counted in the 1-year period
[c]We do not report days at home data for the 0.03% of participants who died within 24 hours of their hip fracture (n=6,362), as these all had zero days at home, by definition
[d]Adjustment was performed using regression-based marginal standardization
SD = standard deviation; SNF = skilled nursing facility; LTC = long-term care

© 2026 Rodin R et al. *JAMA Network Open*.

eTable 3. Multivariable Regression Model of Proportion of Days Alive at Home at 1-Year After Hip Fracture Among Decedents With Dementia[a]

		Model 1[b]		Model 2[c]		Model 3[d]	
	Proportion of DAH, unadjusted Mean (SD)	Change in DAH Proportion, adjusted mean	Proportion of DAH, adjusted mean	Change in DAH Proportion, adjusted mean	Proportion of DAH, adjusted mean	Change in DAH Proportion, adjusted mean	Proportion of DAH, adjusted mean
**Age**							
**<75**	0.64 (0.34)	Ref.	0.64	Ref.	0.65	Ref.	0.65
**75-84**	0.62 (0.35)	-0.02	0.62	-0.03	0.62	-0.03	0.62
**≥85**	0.61 (0.36)	-0.02	0.61	-0.04	0.61	-0.04	0.61
**Sex**							
**Male**	0.61 (0.34)	Ref.	0.61	Ref.	0.60	Ref.	0.60
**Female**	0.62 (0.36)	0.01	0.62	0.02	0.62	0.02	0.62
**Race**							
**Non-Hispanic White**	0.61 (0.36)	Ref.	0.61	Ref.	0.61	Ref.	0.61
**American Indian/Alaska Native**	0.61 (0.34)	-0.00	0.61	0.03	0.63	0.03	0.63
**Asian/Pacific Islander**	0.64 (0.35)	0.03	0.64	0.03	0.64	0.03	0.64
**Black/African American**	0.63 (0.32)	0.02	0.62	0.05	0.66	0.05	0.66
**Hispanic**	0.70 (0.32)	0.09	0.70	0.09	0.70	0.09	0.70
**Other**	0.68 (0.33)	0.07	0.68	0.07	0.67	0.07	0.67
**Unknown**	0.60 (0.34)	-0.02	0.59	-0.01	0.60	-0.01	0.60
**Region**							
**East North Central**	0.58 (0.36)			Ref.	0.58	Ref.	0.58
**East South Central**	0.62 (0.35)			0.04	0.62	0.04	0.62
**Mid-Atlantic**	0.56 (0.36)			-0.03	0.55	-0.03	0.55
**Mountain**	0.72 (0.32)			0.13	0.71	0.13	0.71
**New England**	0.51 (0.38)			-0.05	0.53	-0.05	0.53
**Pacific**	0.73 (0.31)			0.13	0.71	0.13	0.71
**South Atlantic**	0.62 (0.34)			0.055	0.63	0.04	0.63
**West North Central**	0.51 (0.38)			-0.06	0.52	-0.06	0.52
**West South Central**	0.62 (0.34)			0.04	0.62	0.04	0.62
**Urban/Rural**							
**Urban**	0.63 (0.35)			Ref.	0.62	Ref.	0.62
**Rural**	0.53 (0.37)			-0.05	0.57	-0.05	0.57
**Medicare Enrollment Status**							

© 2026 Rodin R et al. *JAMA Network Open*.

**Fee-for-Service**	0.58 (0.34)			Ref.	0.58	Ref.	0.59
**Medicare Advantage**	0.69 (0.36)			0.10	0.68	0.10	0.68
**Medicaid Eligibility**							
**No**	0.65 (0.34)			Ref.	0.65	Ref.	0.65
**Yes**	0.48 (0.38)			-0.19	0.46	-0.19	0.46
**Elixhauser score**							
**Q1 (-26 – 0)**	0.62 (0.36)					Ref.	0.61
**Q2 (1-8)**	0.63 (0.35)					0.01	0.62
**Q3 (9-15)**	0.61 (0.36)					-0.00	0.61
**Q4 (16-81)**	0.61 (0.34)					0.00	0.62
**Delirium during the admission**							
**No**	0.62 (0.35)					Ref.	0.62
**Yes**	0.60 (0.35)					-0.02	0.60
**Fracture location**							
**Femoral neck**	0.63 (0.35)					Ref.	0.63
**Intertrochanteric**	0.61 (0.35)					-0.02	0.61
**Subtrochanteric**	0.57 (0.35)					-0.06	0.57
**Unspecified**	0.61 (0.35)					-0.02	0.61
**Surgical Procedure**							
**Total arthroplasty**	0.63 (0.35)					Ref.	0.61
**Hemiarthroplasty**	0.62 (0.35)					-0.00	0.61
**Internal Fixation/Open Reduction**	0.60 (0.35)					0.00	0.61
**Non-operative**	0.66 (0.36)					0.02	0.63

[a]Decedents in this model included those who died >30 days after hip fracture.
[b]Model 1 means are adjusted for age, sex, and race and ethnicity
[c]Model 2 means are adjusted for all covariates in Model 1 and insurance status (Medicare Advantage enrollment and Medicaid eligibility), geographic region, and rurality
[d]Model 3 means are adjusted for all covariates in Model 2 and comorbidities, delirium during index admission, fracture type, and procedure type
SD = standard deviation; DAH = days at home; Q = quartile

© 2026 Rodin R et al. *JAMA Network Open*.

eTable 4. Sensitivity Analysis of Multivariable Regression Model of Days Alive at Home at 1-Year After Hip Fracture Among Older Adults With Dementia, Restricted to Fee-For-Service Beneficiaries

		Model 1[a]		Model 2[b]		Model 3[c]	
	DAH, unadjusted mean[1] (SD)	Change in DAH, adjusted mean[1]	DAH, adjusted mean[1]	Change in DAH, adjusted mean[1]	DAH, adjusted mean[1]	Change in DAH, adjusted mean[1]	DAH, adjusted mean[1]
**Age**							
**<75**	261.3 (121.3)	Ref.	260.4	Ref.	269.1	Ref.	268.2
**75-84**	256.8 (123.6)	-3.9	256.6	-12.1	257.0	-11.6	256.6
**≥85**	243.6 (131.1)	-16.6	243.9	-27.2	241.9	-25.9	242.3
**Sex**							
**Male**	255.5 (121.8)	Ref.	254.2	Ref.	251.1	Ref.	251.4
**Female**	248.0 (129.6)	-5.7	248.4	-2.0	249.1	-2.5	249.0
**Race**							
**Non-Hispanic White**	248.3 (128.6)	Ref.	248.4	Ref.	246.3	Ref.	246.3
**American Indian/Alaska Native**	253.4 (128.1)	3.3	251.7	21.9	268.2	22.4	268.7
**Asian/Pacific Islander**	262.7 (122.8)	15.2	263.6	27.2	273.5	27.1	273.4
**Black/African American**	269.1 (125.8)	-0.4	248.0	17.1	263.4	16.9	263.2
**Hispanic**	270.2 (117.2)	21.2	269.7	40.8	287.2	41.0	287.3
**Other**	267.9 (116.5)	17.8	266.2	21.1	267.4	20.9	267.2
**Unknown**	261.1 (124.2)	8.2	256.6	21.0	267.4	20.9	267.2
**Region**							
**East North Central**	236.9 (131.5)			Ref.	237.0	Ref.	237.3
**East South Central**	250.1 (129.3)			15.5	252.5	15.2	252.6
**Mid-Atlantic**	238.1 (132.0)			1.5	238.5	1.0	238.4
**Mountain**	276.4 (112.2)			36.1	273.1	35.5	272.8
**New England**	228.3 (139.8)			-4.9	232.1	-5.4	231.9
**Pacific**	276.7 (107.7)			39.4	276.4	38.9	276.3

© 2026 Rodin R et al. *JAMA Network Open*.

**South Atlantic**	257.3 (122.5)			18.9	255.9	18.6	255.9
**West North Central**	213.2 (145.9)			-21.7	215.4	-22.1	215.3
**West South Central**	252.6 (128.7)			13.3	250.3	13.0	250.4
**Urban/Rural**							
**Urban**	255.0 (124.2)			Ref.	253.5	Ref.	253.6
**Rural**	227.2 (140.0)			-20.1	233.4	-20.5	233.1
**Medicaid Eligibility**[d]							
**No**	261.8 (119.3)			Ref.	263.4	Ref.	263.3
**Yes**	196.7 (149.3)			-75.0	188.4	-74.3	189.0
**Elixhauser score**[e]							
**Q1 (-28 – 0)**	255.9 (126.5)					Ref.	255.3
**50.6 (128.7)**					-5.6	249.7	
**Q3 (9-16)**	248.9 (128.3)					-7.0	248.3
**Q4 (17-87)**	243.1 (127.8)					-10.8	244.5
**Delirium during the admission**							
**No**	250.1 (128.1)					Ref.	250.1
**Yes**	247.1 (126.5)					-4.4	245.7
**Fracture location**							
**Femoral neck**	258.2 (125.5)					Ref.	257.1
**Intertrochanteric**	244.7 (129.0)					-12.1	245.0
**Subtrochanteric**	238.0 (129.0)					-18.5	238.6
**Unspecified**	244.0 (130.3)					-13.1	244.1
**Surgical Procedure**							
**Total arthroplasty**	272.4 (116.0)					Ref.	261.4
**Hemiarthroplasty**	252.7 (127.9)					-14.6	246.9
**Internal Fixation/Open Reduction**	246.4 (128.2)					-11.8	249.7
**Non-operative**	252.8 (129.5)					-5.4	256.1

[a] [a]Model 1 means are adjusted for age, sex, and race and ethnicity
[b]Model 2 means are adjusted for all covariates in Model 1 and insurance status (Medicare Advantage enrollment and Medicaid eligibility), geographic region, and rurality

© 2026 Rodin R et al. *JAMA Network Open*.

[c]Model 3 means are adjusted for all covariates in Model 2 and comorbidities, delirium during index admission, fracture type, and procedure type
[d]Enrollment in Medicare Advantage was not included as a covariate because this analysis only included fee-for-service beneficiaries
[e]Elixhauser comorbidity index scores utilized MedPAR, Outpatient and Carrier files for this sensitivity analysis restricted to fee-for-service beneficiaries
SD = standard deviation; DAH = days at home; Q = quartile

© 2026 Rodin R et al. *JAMA Network Open*.

eReferences

1. Adler RR, Xiang L, Shah SK, et al. Hip Fracture Treatment and Outcomes Among Community-Dwelling People Living With Dementia. *JAMA Network Open*. 2024;7(5):e2413878-e2413878. doi:10.1001/jamanetworkopen.2024.13878
2. Kim DH, Lee J, Kim CA, et al. Evaluation of algorithms to identify delirium in administrative claims and drug utilization database. *Pharmacoepidemiol Drug Saf*. Aug 2017;26(8):945-953. doi:10.1002/pds.4226
3. Alexander N. What's more general than a whole population? *Emerg Themes Epidemiol*. 2015;12:11. doi:10.1186/s12982-015-0029-4
4. Redelings MDS, Frank.; Smith, Lisa V.; Greenland, Sander. Why Confidence Intervals Should be Used in Reporting Studies of Complete Populations. *The Open Public Health Journal*. 2012;5:52-54.
5. Graubard BI, Korn EL. Inference for superpopulation parameters using sample surveys. *Statistical Science*. 02/01 2002;17:73-96.

© 2026 Rodin R et al. *JAMA Network Open*.
