# Supplementary material for: Days at Home After Hip Fracture Among Older Adults With and Without Dementia
Source: JAMA Netw Open. 2026 Jun 16;9(6):e2618658. doi: 10.1001/jamanetworkopen.2026.18658 (PMC13273490; doi:10.1001/jamanetworkopen.2026.18658)
Supplement: Supplement 2. — Data Sharing Statement [file jamanetwopen-e2618658-s002.pdf]

Data Sharing Statement

Rodin. Days at Home After Hip Fracture Among Older Adults With and Without Dementia. *JAMA Netw Open*. Published June 16, 2026. doi:10.1001/jamanetworkopen.2026.18658

Data
Data available: No

Additional Information
Explanation for why data not available: This study used Medicare claims data provided by the Center for Medicare and Medicaid Services (CMS), accessed via their Virtual Research Data Center. We are unable to share data with researchers directly as doing so would violate the terms of our data use agreement. Researchers interested in using our analytic dataset for a replication study or other analysis may access the data through a data use agreement with CMS, which would provide the data directly.
